# Chronovaccination: Harnessing circadian rhythms to optimize immunisation strategies

**DOI:** 10.3389/fimmu.2022.977525

**Published:** 2022-10-07

**Authors:** Claire O. Otasowie, Rachel Tanner, David W. Ray, Jonathan M. Austyn, Brendon J. Coventry

**Affiliations:** ^1^ Green Templeton College, University of Oxford, Oxford, United Kingdom; ^2^ Wolfson College, University of Oxford, Oxford, United Kingdom; ^3^ The Jenner Institute, Nuffield Department of Medicine, University of Oxford, Oxford, United Kingdom; ^4^ Institute of Human Sciences, University of Oxford, Oxford, United Kingdom; ^5^ National Institute for Health Research (NIHR) Oxford Biomedical Research Centre, John Radcliffe Hospital, Oxford, United Kingdom; ^6^ Oxford Centre for Diabetes, Endocrinology and Metabolism, University of Oxford, Oxford, United Kingdom; ^7^ Nuffield Department of Surgical Sciences, University of Oxford, John Radcliffe Hospital, Oxford, United Kingdom; ^8^ Department of Surgery, University of Adelaide, Royal Adelaide Hospital, Adelaide, SA, Australia

**Keywords:** chronovaccination, circadian rhythm, diurnal, chronobiology, vaccination, vaccine, innate & adaptive immunity, immune response

## Abstract

Vaccination, as a public health measure, offers effective protection of populations against infectious diseases. Optimising vaccination efficacy, particularly for higher-risk individuals, like the elderly whose immunocompromised state can prevent the development of robust vaccine responses, is vital. It is now clear that 24-hour circadian rhythms, which govern virtually all aspects of physiology, can generate oscillations in immunological responses. Consequently, vaccine efficacy may depend critically on the time of day of administration(s), including for Covid-19, current vaccines, and any future diseases or pandemics. Published clinical vaccine trials exploring diurnal immune variations suggest this approach could represent a powerful adjunct strategy for optimising immunisation, but important questions remain to be addressed. This review explores the latest insights into diurnal immune variation and the outcomes of circadian timing of vaccination or ‘chronovaccination’.

## Introduction

Vaccination is a powerful and cost-effective public health strategy to protect against infectious diseases, with ~2-3 million lives saved yearly through global infant immunisation programmes (1, 2). However, heterogeneity in vaccine responses remains problematic, including vaccine failure, due to individual factors such as age, genetic background and overall health and immune status (3). The elderly are of particular interest, since age-associated immune decline and chronic illness comorbidity increase susceptibility to infection, with potentially less effective vaccine responses (3, 4). Hence, new strategies to optimise vaccine-induced immunity are needed. One innovative approach may be to exploit the impact of circadian rhythms on immune responses, by controlling the timing of vaccination to enhance protective immunity through ‘chronovaccination’.

## Circadian clock control

**Figure 1:**
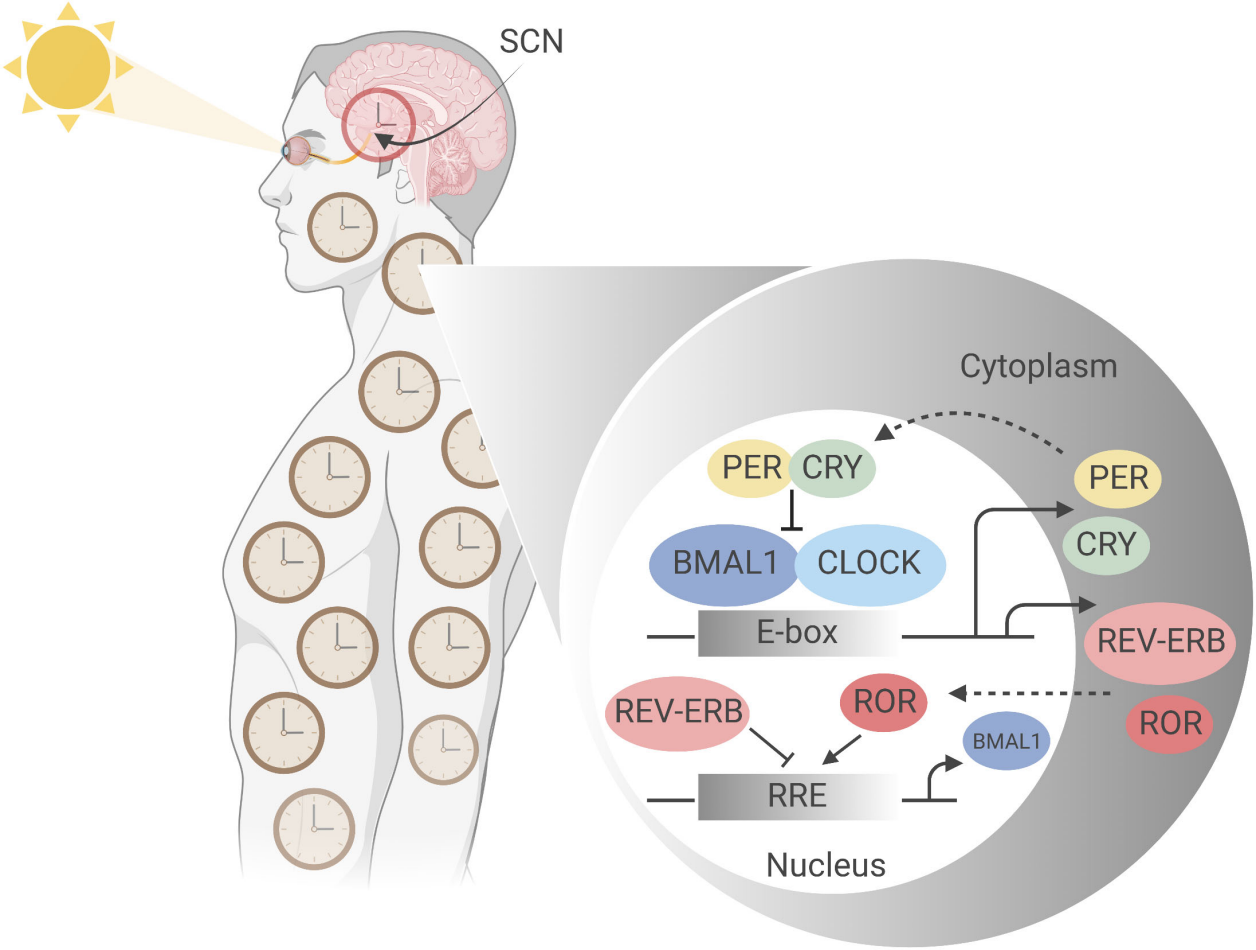
Molecular mechanism of the mammalian circadian clock. The mammalian circadian clockwork comprises a set of conserved, self-sustaining transcriptional- translational feedback loops that begin at ‘dawn’ with transcription factors CLOCK and BMAL1, the positive regulators of the central loop. CLOCK and BMAL1 heterodimerize and bind E-box regulatory elements to drive the expression of target genes including Period (Per) and Cryptochrome (Cry) clock genes (5). Accumulating in the cytoplasm over the course of the circadian day, negative regulators PER and CRY, having complexed to form PER-PER homo- or PER-CRY heterodimers, translocate into the nucleus where they inhibit CLOCK : BMAL1 activity at E-box sites, thereby suppressing their own synthesis (6, 7). Subsequent PER and CRY degradation at ‘night’ allows resetting of the clock, and CLOCK : BMAL1 activity to resume. This generates stable oscillations in target gene expression with a period of approximately 24 hours (8). PER and CRY stability is thus integral to circadian periodicity: mutations affecting their phosphorylation state, for example, dramatically alter period length, as first demonstrated by Menaker’s tau mutant hamster, which is a gain of function mutation in CK1ε (9). A second feedback loop comprises CLOCK and BMAL1 driving expression of nuclear receptors RORα (NR1F1), REV-ERBα (NR1D1) and its paralogue REV-ERBβ (NR1D2), which act *via* ROR-response elements (RORE) in the proximal Bmal1 promotor to modulate Bmal1 expression; RORα as a transactivator and the REV-ERBs as transrepressors (5). Figure created with BioRender.com.

Pioneering 2017 Nobel Prize in Physiology or Medicine recipient chronobiologists Hall, Robash and Young, elucidated the mechanistic basis of circadian rhythms (**Figure 1**) (10). Indeed, circadian rhythms govern virtually all elements of our physiology, including the immune system (11). The temporal organisation of physiology is driven by environment and behaviour, and also by cell-intrinsic circadian oscillators (12). Discoveries made under highly controlled conditions have identified autonomous circadian rhythms. However, in the real-world, analysis is complicated by environmental factors such as light-dark and behavioural rhythms, including sleep-wake and feeding times (13, 14).

In humans, the central circadian clock is located in the suprachiasmatic nucleus (SCN) of the hypothalamus, where it receives information on light exposure *via* the retinohypothalamic tract, a non-image forming mechanism of ‘entrainment’ which results in synchronisation of the internal clock to external time cues (12, 15, 16) (**Figure 1**). However, most body cells have intrinsic circadian oscillators which are entrained to the SCN by autonomic and humoral pathways, often indirectly regulated as a result of timed eating (12). Approximately 50% of the expressed genes vary through circadian time, with up to 40% of metabolic pathways showing 24-hour periodic oscillations (17–19). Importantly, most immune system cells retain intrinsic circadian rhythmicity, with impacts documented on immunological outputs such as the relative size and functions of immune cell populations, inflammatory responses, and responses to infection. It is no surprise that some studies have identified a role for timing in immune responses to vaccination (11).

## Circadian rhythms and the immune response

There is increasing evidence for tight, bidirectional crosstalk between the circadian and immune systems, resulting in temporal gating of the magnitude of immunological responses (11, 20). Rhythmicity in innate immune functions has been long established (11, 21–23). Recent molecular evidence has similarly shown that adaptive immunity is under circadian control. Intrinsic circadian oscillator existence in T-cells was first demonstrated in 2011 by clear rhythmicity of immune-gene (IFNγ, CD40L) and clock-gene expression in polyclonally- stimulated CD4+ T-cells isolated from human blood over a 24-hour period (24). This discovery was soon complemented with the first description of circadian oscillations in B-cells from mice housed in 12:12 light:dark conditions or in constant darkness (25). Clock gene expression in CD8+ T-cells has yet to be formally demonstrated, although it is known that rhythmicity in their antigen-specific proliferative responses requires a functional intrinsic clock (26).

A comprehensive analysis of circadian clock influence on the immune system is reviewed elsewhere (11). However, leukocyte migratory behaviour, which is critical for induction of robust adaptive (T-cell and/or B-cell) responses, is of particular interest. In 2017, a seminal mouse study demonstrated that the cellularity of lymph and lymph nodes (LN) oscillates throughout the day, being highest during the behavioural ‘active phase’ of the organism (at night for mice, and day for humans) due to cell-intrinsic rhythmic expression of critical retention and egress factors (27). Consistent with lymphocyte recirculation dynamics, naïve CD4+ and CD8+ T-cell counts in human blood peak at night (02:00) and decline during daytime to an early afternoon (14:00) nadir (28, 29). Dendritic cell (DC) migration into lymph nodes shows similar evidence of rhythmicity due to cell autonomous clocks acting in both DC and lymphatic endothelial cells (30). Even the expression and function of molecules such as Toll-like receptor 9 (TLR9), ligation of which can induce DC migration, is regulated in a temporal manner (31).

Several lines of evidence indicate that the outcome from infection is determined by the time of day at which it is initiated in a clock gene-dependent manner. Infection of mice with *Salmonella typhimurium* during their rest phase, for example, resulted in higher bacterial loads compared with those infected in the middle of the active phase, and this is CLOCK-dependent (32). Furthermore, *Bmal1* (**Figure 1**) in monocytes can modulate *Leishmania* parasite burden (33); and herpes, influenza A, RSV and *Paramyxoviridae* respiratory virus infections are enhanced when *Bmal1* is disrupted (34, 35). These and other studies (36) provide functionally-relevant *in vivo* evidence that the circadian clock can regulate pathogen-specific immunity; further evidence suggests it also regulates disease severity in experimental studies of inflammatory diseases (31).

## Chronovaccination

From the foregoing, it is clear there are strong theoretical and experimental lines of evidence supporting potentially critical roles for timing in immunisation protocols. Making therapeutic use of the link between chronobiology and immunity is a novel concept that is rapidly gaining attention for its potential to improve drug and vaccine delivery and efficacy (37, 38). We have coined the term ‘chronovaccination’ for chronotherapy applied in the context of vaccines, to embrace emerging evidence suggesting that adjusting the time-of-day of vaccination to align with the optimal circadian phase could be a powerful tool to maximise vaccine immunogenicity.

Excitingly, taking biological timing of vaccination into consideration might represent a simple, cost-effective complementary approach to increasing vaccine immunogenicity alongside other considerations in vaccine design. This is particularly relevant given the slow pace of adjuvant discovery, partly due to safety concerns (39), or where behavioural interventions, like stress reduction or exercise before vaccination, are impractical (40).

**Figure 2:**
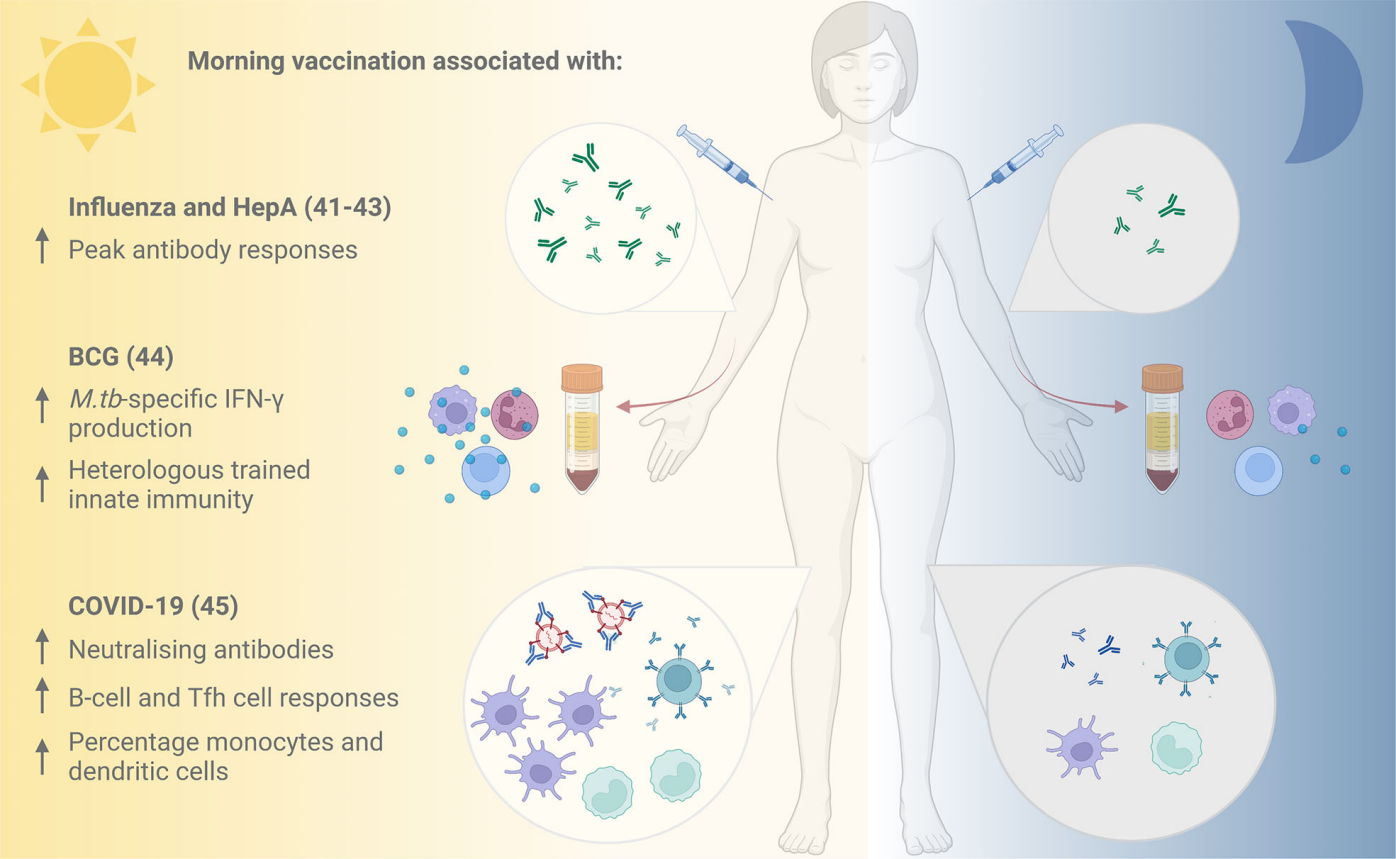
Evidence for improved vaccine responses following morning compared with afternoon vaccination. Vaccination against influenza and hepatitis A in the morning has been associated with increased peak antibody responses (41), and morning influenza vaccination with higher specific antibody responses to the A/H1N1 influenza strain (42, 43). Cells taken following morning BCG vaccination demonstrated elevated M.tb-specific IFN-γ production and stronger heterologous innate immune responses *in vitro* compared with those taken following afternoon vaccination (44). An inactivated COVID-19 vaccine induced higher neutralising antibody titres, improved B-cell and Tfh cell responses and higher percentages of monocytes and dendritic cells when administered in the morning (45). HepA, Hepatitis A; BCG, Bacille Calmette Guérin; *M.tb, Mycobacterium tuberculosis*; IFN-γ, interferon-gamma; Tfh, T follicular helper. Figure created with BioRender.com.

The new appreciation for circadian orchestration of immune responses is translating into promising clinical chronovaccination research (**Figure 2**). Leading the efforts, in 2016, Long and colleagues (42, 43) performed the first large-scale randomised controlled trial (RCT) assessing the effect of different vaccination times: UK adults aged over 65 received seasonal influenza vaccines either in the morning (9:00-11:00) or afternoon (15:00-17:00). One month later, significantly higher specific antibody responses were mounted against the A/H1N1 and B influenza strains (but not the A/H3N2 strain) after vaccination in the morning compared to the afternoon (42, 43).

A beneficial influence of morning vaccination is further supported by subsequent trials. In 2020, de Bree and others (44) assessed the effect of time of vaccination on specific and non-specific immunity induced by the only licenced TB vaccine, Bacillus Calmette-Guérin (BCG). When healthy adults from the Netherlands (mean age 26 years) received the BCG vaccine in the morning (08:00-09:00) or evening (18:00-18:30), *Mycobacterium tuberculosis (M.tb)*-specific IFN-γ production by cells *in vitro* was elevated at 3 months post- vaccination in the group vaccinated in the morning, but not in the evening (44). Furthermore, BCG vaccination in the morning induced stronger trained innate immune responses (‘innate memory’) against the heterologous pathogen *Staphylococcus aureus* (44). Given growing interest in the potential non-specific effects of BCG and other vaccines in protecting against all-cause mortality, this could have broader-reaching implications for vaccine effectiveness.

Most recently, there are early indications that morning vaccination has a positive influence on the neutralising antibody response to an inactivated vaccine against SARS-CoV-2 from a prospective cohort study of healthcare workers in China (45). Participants vaccinated in the morning had improved B-cell and T follicular helper (Tfh) cell responses at 8 weeks post-vaccination, and higher percentages of circulating monocytes and dendritic cells. An observational study of UK healthcare workers found that time of day of vaccination with the Pfizer mRNA or Oxford-AstraZeneca adenoviral SARS-CoV-2 vaccines was one of several factors influencing the magnitude of anti-spike antibody responses induced (46).

While the mechanism by which time of day influences vaccine-induced immunity remains unclear, studies have considered the role of circulating steroid hormones which vary diurnally and have been associated with immunoregulation and vaccine responses (47–50). However, the time-of-day effects on antibody response to influenza vaccination did not appear to be mediated by these hormones (42, 43). Similarly, de Bree et al. found no associations between circulating cortisol levels and cytokine production *in vitro* following specific or non-specific stimulation of cells from BCG vaccinated volunteers (44). Previous studies have indicated a diurnal effect on BCG-induced immune trafficking including neutrophil migration (51–53), which may be relevant given the central role for neutrophils in transferring BCG to the draining LNs (54).

Rather than circadian rhythms in soluble factors in the circulation, peripheral molecular clocks in cells (coordinated by the central clock in the SCN of the hypothalamus) may play a key role in timed vaccine effects. Indeed, circadian clock genes have been shown to oscillate in immune cells (24). De Bree et al. hypothesise that a molecular intrinsic clock within monocytes and their progenitors, neutrophils or hematopoietic stem cells could contribute to the observed effects of time of day of vaccination on BCG-induced trained immunity (44). It is similarly possible that clocks in other peripheral blood mononuclear cells and vascular and lymphatic endothelial cells play a role in time of day influences on vaccine responses, but further work is required to understand this association.

Interestingly, epigenetic differences resulting in increased chromatin accessibility in genes important for the mTOR pathway at 3-months post-BCG vaccination have been identified in morning- but not evening-vaccinated participants. Morning-vaccinated individuals also showed enrichment of transcription factors involved in mTOR signalling and associated with active histone marks (44). As the mTOR pathway is key to the induction of trained innate immunity, this may in part account for diurnal variation in the non-specific effects of BCG vaccination (55).

Future studies to validate these early findings might include high-dependency, frailer populations to investigate whether chronovaccination remains effective despite more complex health profiles (56). It is clear that further functional studies are also needed to clarify the molecular and cellular mechanisms involved. Nonetheless, these timely clinical trials provide important preliminary evidence that chronovaccination may be effective for improving the protective response to vaccination, particularly in the elderly.

## Population age and chronovaccination outcomes

Evidence for a diurnal response to vaccination is less convincing in younger adults. The literature for this demographic is largely inconclusive: some investigators suggest time-of-day dependent effects on post-vaccination antibody responses (41, 57), while others find no difference (58, 59). This apparent inconsistency between age groups is perhaps explained (60) by reports that humans show heterologous chronotypes in circadian clock timing (sleep/wake) even under entrained conditions, exposing genetic and environmental complexity. Hence, circadian phases are variable across a population, being subject to both internal and external influences.

In 2016, three landmark genome-wide association study (GWAS) analyses of self-reported chronotype (61–64) identified multiple significant genetic associations, highlighting the complex and individual nature of circadian timing. Human populations are typically ‘masked’, referring to people following external cues and living against their endogenous circadian clock; such people experience circadian misalignment. A prevalent, and extreme cause of circadian misalignment is shiftwork. Interestingly, such individuals have independently increased risk of immune disorders such as asthma (65), cardiovascular disease (66, 67), type-2 diabetes (67), breast cancer (68), Covid-19 infection (69), hospitalisation and reduced vaccination efficacy (69, 70), indicating the potentially serious effects of circadian disruption.

There are highly systematic age-dependent changes of chronotype (60). Older adults become phase advanced (60) perhaps owing to greater morning exposure to natural bright light (71), which is widely accepted as the strongest zeitgeber (i.e. entraining stimulus) (14). In contrast (72), delayed chronotypes (‘late-risers’) were found in a university student population aged 17-26 years (60). Potentially, dramatic circadian phase differences could confound data analysis, making comparisons of chronovaccination outcomes difficult across different age groups. Surprisingly, individual chronotype has been largely overlooked as a variable in chronovaccination studies to date. These potentially confounding inter-participant phase differences need consideration in future studies for vaccination timing between older and younger adults.

Beyond circadian *phase*, many studies document a decline in circadian *amplitude* with age (73). Reduced immune responses are shown by higher mortality in >65 year old patients, for example for influenza, Covid-19, bacterial pneumonia (74), and progressively reduced responses to Covid vaccinations (75). Some new intriguing mechanistic insights show diurnal rhythm and some innate immune responses in murine macrophages are abolished with ageing (73).

It is possible that chronovaccination, like other unconventional approaches that seek to optimize the circumstances surrounding vaccination, is only effective for ‘sub-optimal’ vaccination responses, as are typical in older age (74). This controversial viewpoint from Edwards et al. (40) suggests that acute exercise only enhanced the antibody response in healthy young adults (mean age 22 years) when participants were given a weakly-immunogenic half-dose of pneumococcal vaccine, as opposed to the full-dose (40). Strengthening this hypothesis will require evidence that non-uniform vaccine responses to behavioural interventions segregate with participant immunocompetence. An informative approach could include a group of participants taking immunosuppressant medication, for example therapeutic glucocorticoids.

Arguably, interventions optimising vaccine immunogenicity should prioritise those where vaccine failure poses the greatest concern, particularly those aged over 65 and the immunocompromised (75–77). Such strategies must account for inflammageing and immunosenescence as two essential characteristics of ageing (74–76). Previous studies have documented shifts in immune cell populations as people age: for example, there is a relative predominance of memory and effector T-cells over naïve T-cells in the elderly (77). Similar changes have been observed for naïve B-cells, diversity of the B-cell repertoire and B-cell production with age (78). Further studies are clearly needed to clarify the impact of population age on outcomes of timed vaccinations.

## Clinical efficacy of chronovaccination

Despite displays of statistical significance in terms of immunogenicity (41–43, 57), elucidating the clinical significance of chronovaccination for vaccine efficacy has received remarkably little attention. It remains largely unknown how, or indeed if, the observed increases in early post-chronovaccination antibody titre and other immune parameters correspond to a better quality of response and, crucially, whether this confers greater protection against infection and/or disease over time. Critics question whether time-of-day of vaccination can truly affect a slowly mounted adaptive response that fundamentally depends on chance and dynamic interactions between highly motile cells (79, 80). This viewpoint is incompatible with the abundant evidence that coordinated adaptive immune responses and outcomes of infection depend also on biological time (11). Evidence of an increase in specific neutralising antibodies [which are broadly predictive of immune protection from symptomatic SARS-CoV-2 infection (81)] following morning compared with evening COVID-19 vaccination could suggest a functionally-relevant influence of the circadian clock (44). Establishing whether the timing of vaccination meaningfully impacts on immunogenicity and clinical protective efficacy from pathogen infections and disease will be crucial before any clear recommendations can be made to change current vaccination practices (80). Indeed, in addition to ‘chronoefficacy’ being noted, so has ‘chronotoxicity’, where deleterious effects might depend on the time-of-day dosing (82).

## Discussion

Chronovaccination may represent a paradigm shift in vaccine immunology. However, pressing questions remain concerning its practicalities. Given inter-individual chronotype heterogeneity, chronovaccination may benefit from a population-specific or personalised approach to accurately predict optimal biological time, such as outlined by Wittenbrink et al. (83) using a single blood sample. The logistical and economic cost of this additional test may, however, pose a barrier to the widespread implementation of ‘individualised’ chronovaccination. Moreover, repeated serial measurements will undoubtedly be required to define the complex immunobiology underlying vaccination responses.

Even sampling itself may be a confounding factor in being able to interpret the outcomes of chronovaccination studies. The fact that peripheral leukocyte circulation shows diurnal oscillations (28) and that innate and adaptive response components can diurnally fluctuate significantly in a vaccinated individual (41–46), makes it perhaps surprising that blood sample ‘collection time-of-day’ is not already considered as a covariate in most chronovaccination trials, and that unvaccinated or placebo control groups are not also included in study designs. For example, differences in baseline number of B-cells (59), or of the capacity of monocytes to produce proinflammatory cytokines (44), vary between samples collected in the morning compared to the evening. Utilising big-data analytics, precise timing of vaccine delivery and sample collection should be recorded in future clinical trials (59).

Chronovaccination protocols may also require tailoring to the vaccine used and the type of protective response required (42, 43). Speculatively, it might emerge that chronovaccination has a different optimal time-of-day for different vaccine dose-strengths, formulations, types, or adjuvants according to their mode of action. Considering the superimposed effects that the sequence of vaccinations may have on their efficacy is important, particularly if chronovaccination is to be applied to infant immunization programmes. Aaby et al. (84) noted in an observational study that oral polio vaccination in the first weeks of life can render the second dose of measles vaccine more effective in reducing overall mortality, and this was further reduced when early BCG vaccination was added (84). This may make the task of accurately evaluating the true clinical impact of chronovaccination even more challenging.

Potentially, chronovaccination offers improved clinical efficacy of current licensed vaccines, particularly those that are partially-effective such as BCG, for the elderly, and to inform the development of novel vaccines and adjuvants. There is strong mechanistic evidence that circadian rhythmicity in immune function influences the innate and adaptive responses according to the time-of-day of initial antigen challenge. However, this is yet to translate into conclusive results from clinical chronovaccination trials due to methodological limitations and insufficient study of the relevance, specifics and generalisability of findings for useful recommendations.

Early reports of increased antibody titre following morning influenza vaccination in the elderly are indeed promising but, crucially, it remains unknown whether the immuno-enhancing effect confers any clinically significant reduction in infection, disease incidence and/or survival. At least, no harm appears evident from morning vaccination. The current evidence does, however, clearly highlight that the time of vaccination and even sample collection can have major (and potentially misleading) effects on study results and correlated outcomes. The most immediate influence of chronovaccination research may lie in its experimental consideration as a covariable in future vaccine clinical trial designs. Further study is certainly needed to resolve the important issues highlighted in this review before any potential future clinical role for chronovaccination can be safely implemented.

In the meantime, it would seem easy and reasonable that ‘time of vaccination’ should be stratified into most vaccine trials for later analysis. ‘Omics’ based deep phenotyping approaches may be key to creating a phase translation map that would aid in identifying translational potential and challenges (85, 86). Indeed, a pilot study has demonstrated the feasibility of characterising the human “chronobiome” at scale (85, 86), and a recent visualisation tool identified rhythms in –omics scale datasets (85–87). Other near-future steps to bridge the chronovaccination knowledge gap may include greater recognition of circadian research within the medical school curricula, vaccinology and immunology courses (as done in the Oxford Masters in Integrated Immunology Course), funding body incentivisation for inclusion in vaccine trial proposals, and industry consideration in the vaccine development pipeline. Although perhaps tempting to speculate that morning vaccination might have higher clinical efficacy, more work is needed to confirm and recommend this safely (82) for general vaccine dosing.

## Author contributions

CO conceived the paper with assistance from JA, partly inspired by the Oxford Masters in Integrated Immunology Course (BC & JA). The first draft was written by CO. RT, DR, BC, and JA contributed to the writing and critical reading of the manuscript. All authors contributed to the article and approved the submitted version.

## Acknowledgments

Figures were created with BioRender.com.

## Conflict of interest

The authors declare that the research was conducted in the absence of any commercial or financial relationships that could be construed as a potential conflict of interest.

## Publisher’s note

All claims expressed in this article are solely those of the authors and do not necessarily represent those of their affiliated organizations, or those of the publisher, the editors and the reviewers. Any product that may be evaluated in this article, or claim that may be made by its manufacturer, is not guaranteed or endorsed by the publisher.
